# Maternal N-Acetyl-Cysteine Prevents Neonatal Hypoxia-Induced Brain Injury in a Rat Model

**DOI:** 10.3390/ijms222413629

**Published:** 2021-12-20

**Authors:** Ola Gutziet, Roee Iluz, Hila Ben Asher, Linoy Segal, Dikla Ben Zvi, Yuval Ginsberg, Nizar Khatib, Osnat Zmora, Michael G. Ross, Zeev Weiner, Ron Beloosesky

**Affiliations:** 1 Department of Obstetrics and Gynecology, Rambam Health Care Campus, Haifa 3525433, Israel; roee.iluz@gmail.com (R.I.); alonhila28@gmail.com (H.B.A.); linoy.sg@gmail.com (L.S.); diklabenzvi@gmail.com (D.B.Z.); yuvalginsberg@gmail.com (Y.G.); khatibnizar@yahoo.com (N.K.); z_weiner@rambam.health.gov.il (Z.W.); ronbel3@gmail.com (R.B.); 2 Ruth and Bruce Rappaport Faculty of Medicine, Technion-Israel Institute of Technology, Haifa 3525408, Israel; 3 Department of Pediatric Surgery, Shamir Medical Center, Tzrifin 7073001, Israel; zmora.osnat@gmail.com; 4 Sackler School of Medicine, Tel Aviv University, Tel Aviv 6997801, Israel; 5 Department of Obstetrics and Gynecology, Harbor-UCLA Medical Center and The Lundquist Institute, Torrance, CA 92270, USA; mikeross@lundquist.org

**Keywords:** hypoxia, brain injury, N-Acetyl Cysteine, inflammation, oxidative stress, sensorimotor dysfunction

## Abstract

Perinatal hypoxia is a major cause of infant brain damage, lifelong neurological disability, and infant mortality. N-Acetyl-Cysteine (NAC) is a powerful antioxidant that acts directly as a scavenger of free radicals. We hypothesized that maternal-antenatal and offspring-postnatal NAC can protect offspring brains from hypoxic brain damage.Sixty six newborn rats were randomized into four study groups. Group 1: Control (CON) received no hypoxic intervention. Group 2: Hypoxia (HYP)-received hypoxia protocol. Group 3: Hypoxia-NAC (HYP-NAC). received hypoxia protocol and treated with NAC following each hypoxia episode. Group 4: NAC Hypoxia (NAC-HYP) treated with NAC during pregnancy, pups subject to hypoxia protocol. Each group was evaluated for: neurological function (Righting reflex), serum proinflammatory IL-6 protein levels (ELISA), brain protein levels: NF-κB p65, neuronal nitric oxide synthase (nNOS), TNF-α, and IL-6 (Western blot) and neuronal apoptosis (histology evaluation with TUNEL stain). Hypoxia significantly increased pups brain protein levels compared to controls. NAC administration to dams or offspring demonstrated lower brain NF-κB p65, nNOS, TNF-α and IL-6 protein levels compared to hypoxia alone. Hypoxia significantly increased brain apoptosis as evidenced by higher grade of brain TUNEL reaction. NAC administration to dams or offspring significantly reduce this effect. Hypoxia induced acute sensorimotor dysfunction. NAC treatment to dams significantly attenuated hypoxia-induced acute sensorimotor dysfunction. Prophylactic NAC treatment of dams during pregnancy confers long-term protection to offspring with hypoxia associated brain injury, measured by several pathways of injury and correlated markers with pathology and behavior. This implies we may consider prophylactic NAC treatment for patients at risk for hypoxia during labor.

## 1. Introduction

Perinatal hypoxia is a major cause of infant brain damage, lifelong neurological disability, and infant mortality [1]. Severe hypoxic ischemic stress may cause cellular energy failure, loss of mitochondrial function, brain edema, and increased release of neurotransmitters and intracellular calcium [1,2,3]. These changes trigger pathological cascades activating microglia, which generate oxidative stress, pro-inflammatory cytokine release, and glutamate toxicity. Together, these factors lead to a vulnerability of pre-oligodendrocytes, especially during the preterm period, with resulting impairment of myelination [4], periventricular leukomalacia, long-term brain injury, and potentially cerebral palsy [5]. Attenuation of maternal and fetal pro-inflammatory responses caused by hypoxic injury may be beneficial in protecting the fetal brain from the initial injury process, and thus from lifelong neurological disability and infant mortality. As of today, there is no treatment that can protect fetal brains from hypoxic damage during and around birth.

N-Acetyl-Cysteine (NAC), a powerful antioxidant, is a precursor in the formation of the antioxidant glutathione. It acts directly as a scavenger of free radicals, especially oxygen radicals. Accordingly, NAC is recommended as a potential treatment option for disorders resulting from generation of free oxygen radicals [6]. NAC has been shown to prevent fetal brain inflammation and brain damage associated with maternal inflammation in a rat model of lipopolysaccharide exposure [7,8]. In humans, antenatal and postnatal NAC treatments were demonstrated to be safe when used in association with maternal chorioamnionitis [9]. In the current study, we hypothesized that maternal antenatal and offspring-postnatal NAC can protect offspring brain from hypoxic brain damage.

## 2. Results

**Figure 1:**
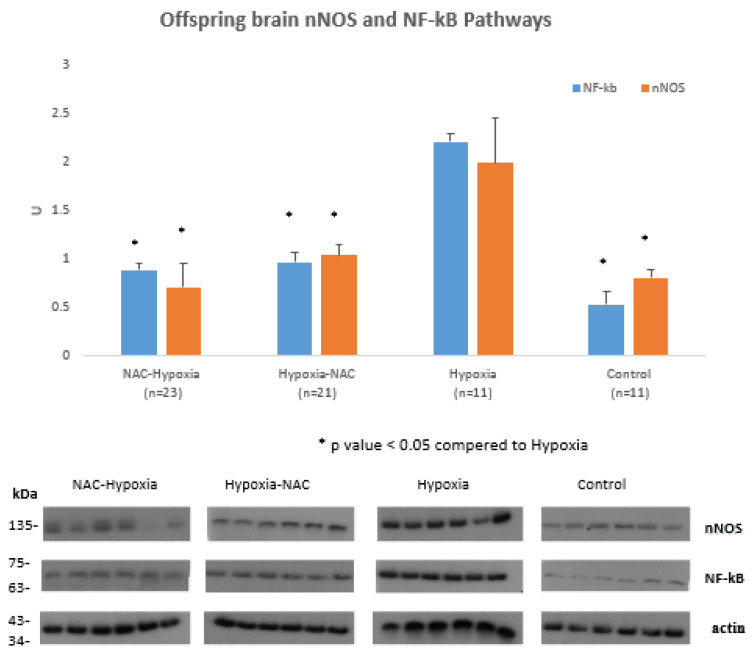
Offspring brain nNOS and NFkB protein levels in Control, Hypoxia, NAC-Hypoxia and Hypoxia-NAC groups: Hypoxia significantly increased offspring brain nNOS and NFkB protein levels compared to control, while NAC treatment decreased hypoxia-induced brain NFkB and nNOS pathways. * Significant difference (*p* < 0.05) compared to Hypoxia.

Offspring brain NFkB, nNOS pathways: HYP pups had significantly increased brain protein levels of NFkB p65 (active form) and nNOS compared to controls (2.20 ± 0.08 vs. 0.53 ± 0.12; 1.99 ± 0.46 vs. 0.80 ± 0.08 U, respectively, *p* < 0.05). NAC administration to HYP offspring (HYP-NAC) resulted in lower brain protein levels of NFkB p65 (active form) and nNOS compared to HYP pups (0.96 + 0.09; 1.03 ± 0.11 U, respectively, *p* < 0.05). NAC treatment during pregnancy prior to hypoxia (NAC-HYP) significantly decreased offspring brain protein levels of NFkB p65 (active form) and nNOS as compared with HYP (0.88 ± 0.06; 0.70 ± 0.25 U, respectively, *p* < 0.05) (Figure 1).

**Figure 2:**
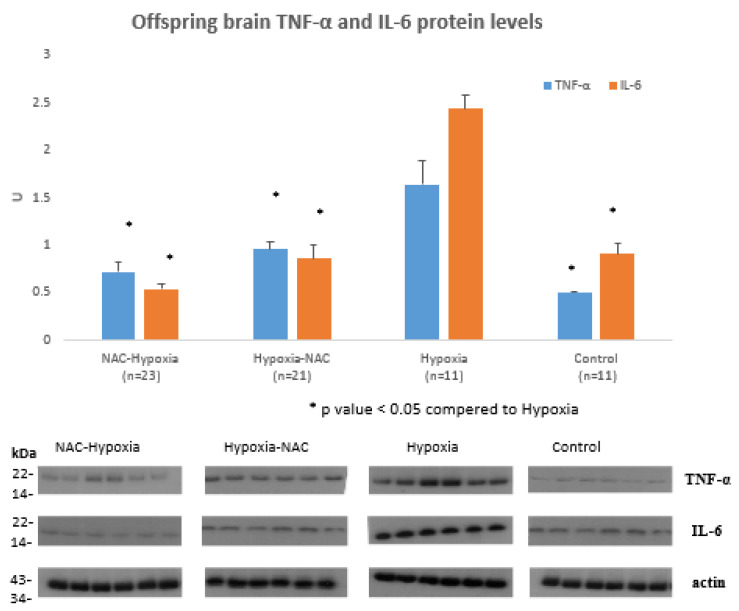
Offspring brain TNF-α and IL-6 protein levels in Control, Hypoxia, NAC-Hypoxia and Hypoxia-NAC groups: Hypoxia significantly increased offspring brain TNF-α and IL-6 protein levels compared to control, while NAC treatment decreased hypoxia-induced brain TNF-α and IL-6 levels. * Significant difference (*p* < 0.05) compared to Hypoxia.

Offspring brain inflammation: HYP pups had significantly increased brain protein levels of TNFα and IL-6 compared to control (1.63 ± 0.25 vs. 0.50 ± 0.01; 2.43 ± 0.14 vs. 0.90 ± 0.11 U, respectively, *p* < 0.05). NAC administration to offspring (HYP-NAC) demonstrated lower brain protein levels of TNFα and IL-6 compared to the HYP group (0.95 ± 0.07; 0.85 ± 0.15 U, respectively, *p* < 0.05). NAC treatment during pregnancy prior to hypoxia (NAC-HYP) significantly decreased offspring brain protein levels of TNFα and IL-6 as compared with HYP (0.71 ± 0.10; 0.53 ± 0.05, respectively, *p* < 0.05) (Figure 2).

**Figure 3:**
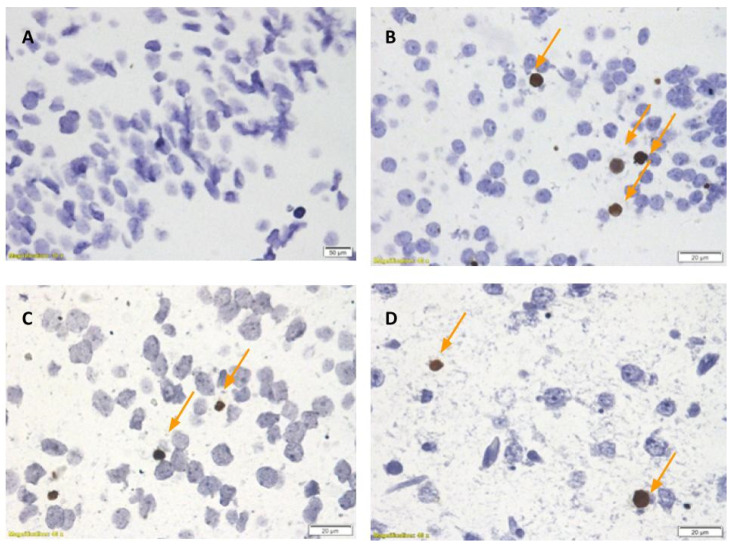
Offspring neuronal Tunel staining (orange arrows) in Control (**A**), Hypoxia (**B**), NAC-Hypoxia (**C**) and Hypoxia-NAC groups (**D**), X40. Hypoxia pups had a higher grade of TUNEL reaction (grade 2) compared to control (grade 1). NAC to pregnant dams or offspring decreased TUNEL reaction to grade 1, similar to controls.

TUNEL staining: HYP pups demonstrated more neuronal apoptosis evidenced by higher grade of brain TUNEL reaction (grade 2) compared to control (grade 1). NAC administration to offspring (HYP-NAC) decreased brain apoptosis to TUNEL reaction grade 1, similar to controls. NAC treatment during pregnancy prior to hypoxia (NAC-HYP) similarly decreased brain apoptosis to TUNEL reaction grade 1 (Figure 3).

**Offspring systemic inflammation:** There was no difference in offspring serum IL6 protein levels among CON, HYP, NAC-HYP, and HYP-NAC groups.

**Figure 4:**
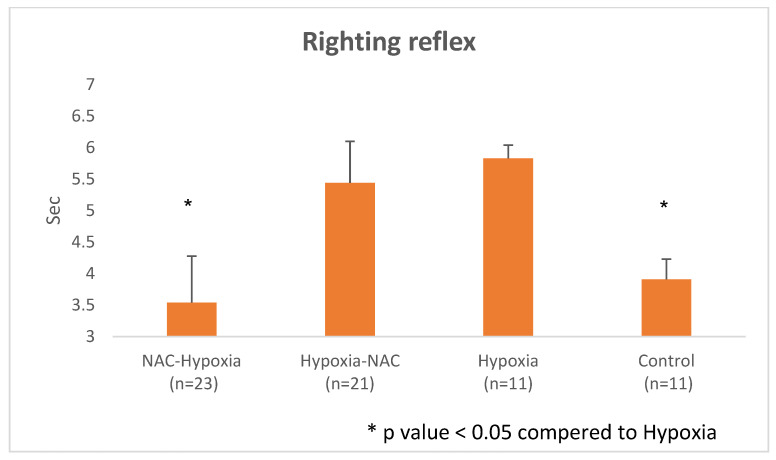
Sensorimotor function evaluation in Control, Hypoxia, NAC-Hypoxia and Hypoxia-NAC groups: Righting reflex on the fifth day of life. Hypoxia significantly Induced acute sensorimotor dysfunction in offspring measured by Righting reflex compared to control. NAC treatment during pregnancy prior to hypoxia significantly attenuated Hypoxia induced acute sensorimotor dysfunction. * Significant difference (*p* < 0.05) compared to hypoxia.

**Sensorimotor dysfunction:** HYP induced acute sensorimotor dysfunction measured by Righting reflex compared to control (5.44 ± 4.2 vs. 3.54 ± 2.01 sec, respectively, *p* < 0.05). NAC treatment during pregnancy prior to hypoxia (NAC-HYP) significantly attenuated HYP induced acute sensorimotor dysfunction (3.91 ± 2.77 vs. 5.44 ± 4.2 sec, respectively, *p* < 0.05) (Figure 4).

**Weight and mortality:** There were no differences among birth weights in the study groups (Table 1). Offspring of NAC treated dams prior to hypoxia (NAC-HYP) weighed significantly more than dams in the HYP group (9.4 ± 0.74 vs. 8.88 ± 0.21 g, respectively, *p* < 0.05) (Table 1). Mortality rates in control, HYP, NAC-HYP, and HYP-NAC groups were 0%, 20% (Day 1 and 2 of life), 4.76% (Day 2 of life), and 8.69% (Day 1 and 3 of life), respectively, but the differences among the groups did not reach statistical significance (Table 1).

There were no birth weight differences in the study groups. Offspring of NAC treated dams weighted significantly more than the HYP group (*p* < 0.05). The difference in mortality rates did not reach statistical significance. * *p* < 0.05 compared to hypoxia.

**Figure 5:**
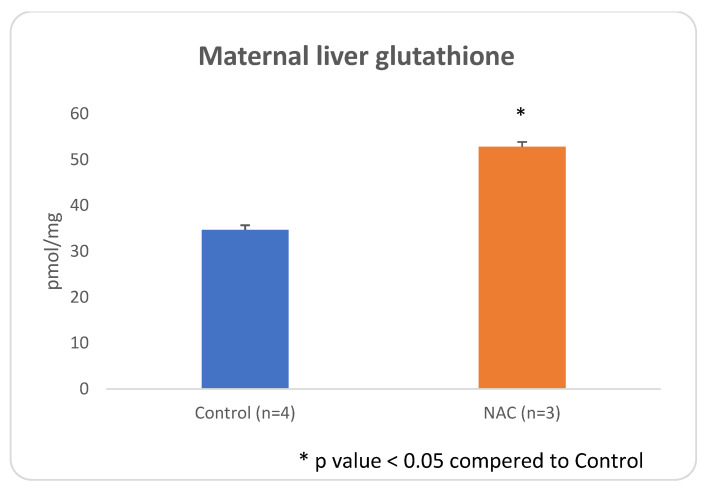
Maternal liver glutathione level in NAC and control group: Maternal NAC significantly increased maternal liver glutathione levels compared to control.* Significant difference (*p* < 0.05) compared to control.

**Maternal liver glutathione level:** Liver glutathione levels in dams treated with NAC intravenously for the last 3 days of pregnancy (n = 3) were significantly higher 5 days after birth, compared to dams treated with NS (n = 4) (52.84 ± 4.12 vs. 34.70 ± 3.56 pmol, respectively, *p* < 0.05) (Figure 5).

## 3. Discussion

We demonstrated that perinatal hypoxic brain damage is mediated by inflammation, NFkB and nNOS pathways, and brain apoptosis as described in other models [10,11,12,13,14,15,16,17]. Further we demonstrated that maternal-antenatal and offspring-postnatal NAC treatment decreased offspring hypoxia-induced brain inflammation, NFkB- and nNOS pathways, and brain apoptosis. We propose that the protective effect is mediated in part by increased liver glutathione levels in dams treated with NAC. Additionally, maternal NAC administration significantly attenuated hypoxia-induced acute sensorimotor dysfunction. In our model, we mimicked the effect of hypoxia on human brain development during the second/third trimester of pregnancy by exposing the rat neonates on day 1–5 of life to hypoxia. The human brain is most vulnerable during this period for long term sequelae [18]. Interestingly the protective effect in the maternal NAC group treatment was evident with administration of NAC to dams in the last 3 days of pregnancy, days before the pups were exposed to hypoxia conditions.

Increase in brain pro-inflammatory cytokines has been associated with adverse neonatal outcomes [15,16]. Increased levels of inflammatory cytokines were demonstrated in brain autopsies of neonates with periventricular white matter damage, a finding correlated with cerebral palsy development later in life [19]. Specifically, IL-6 and TNFα brain levels are associated with long term neurological outcomes in the offspring and are thought to be involved in the pathophysiology of the brain injury [20,21,22,23,24]. In our model, exposure to hypoxia significantly increased the offspring brain protein levels of both IL-6 and TNFα.

We further evaluated pathways associated with oxidative stress and inflammation (i.e., NFkB and nNOS). The free radical nitric oxide (NO) appears to play a critical role in perinatal hypoxic brain injury, as reactive oxygen and nitrogen species are produced in antenatal hypoxic–ischemic insult [25]. NO is synthetized enzymatically from l-arginine by three NO synthase isoforms, iNOS, eNOS and nNOS [26]. nNOS is constitutively expressed in the central nervous system and has been shown to play an important role in physiological functions, such as memory, learning, neurogenesis [27], and long-term regulation of synaptic transmission [27,28,29,30]. nNOS functions include control differentiation, development, apoptosis, and synaptic plasticity in the central neural system. Free radicals and inflammatory processes also may induce secondary damage by activation of NFκB pathway. NFkB activation leads to an increase in gene transcription for pro-inflammatory mediators and diverse functions in the nervous system, including roles in plasticity, learning, and memory [31,32]. In this study, we demonstrated an increase in nNOS active form and NFκB in the offspring brains following exposure to hypoxia. The hypoxic-ischemic insult causes mitochondrial impairment and release of pro-apoptotic proteins, resulting in brain cellular apoptosis [1]. In the present study, we demonstrated hypoxia-induced brain apoptosis by increased TUNEL staining. Hypoxic insult induced neonatal sensorimotor dysfunction (abnormal righting reflex at the first days of life), which correlates significantly with long-term neurofunction assessed at 8 weeks of age [33].

NAC is a recommended treatment for disorders resulting from free oxygen radicals [6]. NAC is a class B drug in pregnancy [9], though the NAC effect on the fetal brain in response to hypoxia was not previously addressed. NAC mechanisms of action include increased glutathione peroxidase levels, antioxidant effect by free-radical scavenging, inhibition of the activation of NF-κB, inhibition of TNF toxicity, NOS inhibition, and prevention of mitochondrial dysfunction [34]. We previously demonstrated that NAC has a protective effect on pups’ brain damage in the Entero Colitis model (NEC) [35]. NEC model is based on pups’ formula feeding thrice daily which induces intestinal inflammation complemented with hypoxia treatment. This work demonstrates that NAC has a protective effect on pups’ brain damage following exposure to hypoxia alone. NAC treatment to the dams days before offspring exposure to hypoxia, or NAC treatment to the offspring following the hypoxia insult, attenuated the inflammatory response in the offspring brain and significantly decreased both IL-6 and TNFα. Similar responses were previously described following NAC administration in rat serum and brain in a model of LPS exposure [7,8,36]. NAC treatment to dams before exposure to hypoxia, or to pups following the hypoxic insult, decreased offspring hypoxia induced brain nNOS and NFκB increase, and decreased offspring hypoxia induced brain apoptosis. NAC administration to dams completely abrogated hypoxia induced acute sensorimotor dysfunction assessed by righting reflex. These findings may be associated with a protective effect of NAC treatment on the long-term neuronal function following hypoxic insult. We demonstrated that liver glutathione levels in dams treated with NAC for the last 3 days of pregnancy were significantly higher than when measured 5 days after birth and after completion of NAC treatment, compared to control.

Oxidative stress is associated with maternal and fetal liver glutathione precursor depletion. Buhimschi et al. [37] demonstrated that maternal NAC elevated liver glutathione, in both dams and fetuses, enabling them to cope with increased oxidative stress. The protective effect of maternal NAC treatment days before offspring exposure to hypoxia was mediated by increased fetal liver glutathione levels. Restoration of maternal, fetal, and newborn oxidative balance by NAC protects the neonatal brain from oxidative brain injury. Our work suggests that maternal NAC treatment prior to perinatal hypoxia exposure or neonatal NAC treatment may protect the offspring brain from hypoxic brain injury.

## 4. Methods

Seven pregnant Sprague–Dawley rats were obtained at day 10 of gestation and allowed to acclimate for a week prior to the beginning of the experiments. Throughout the study, dams were maintained with an alternating 12-h light/dark cycle with access to food and water ad libitum, under constant room humidity and temperature.

**Scheme 1:**
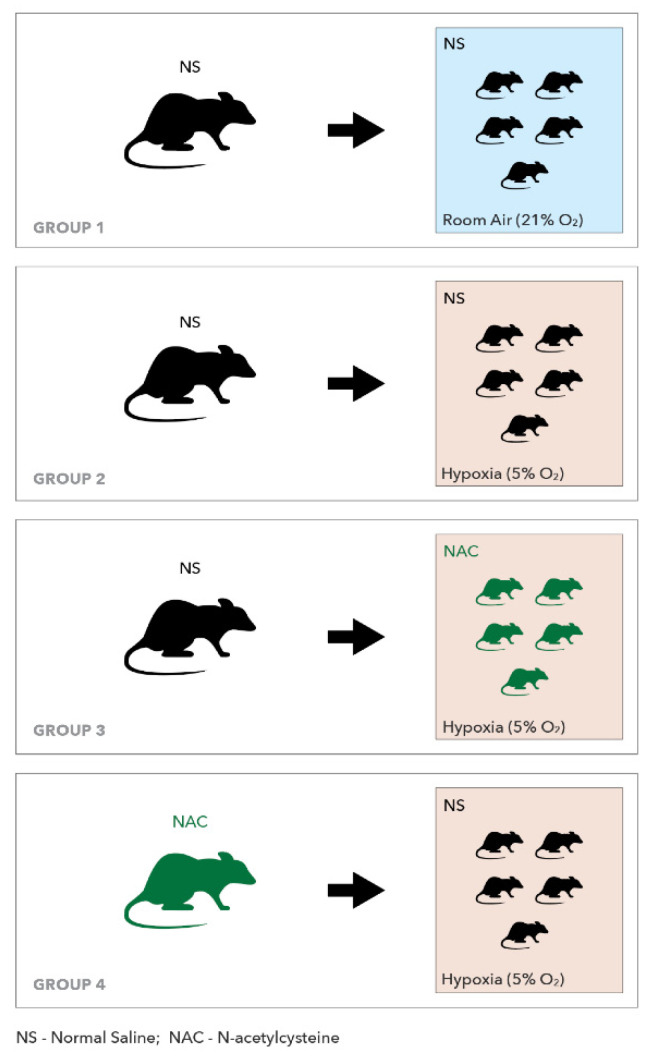
Study groups: Group 1: Control (CON). Group 2: Hypoxia (HYP). Group 3: Hypoxia-NAC (HYP-NAC). Group 4: NAC Hypoxia (NAC-HYP).

The study comprised four groups (Scheme 1). Groups 1–3: pups were randomized from four dams treated with 0.3 mL normal saline (NS) once daily intravenously (Tail) in the last 3 days of pregnancy. Group 4: pups were randomized from three dams treated with N- Acetyl- Cysteine (NAC) 300 mg/kg (Sigma-Aldrich, St. Louis, MO, USA) reconstituted in water intravenously (Tail) in the last 3 days of pregnancy. Following delivery, we adjusted the number of pups to 10–12 in each dam. Group 1 (n = 11): Control (CON) received no hypoxic intervention. Pups received intraperitoneal (IP) injections of NS (100 µL) three times daily, beginning on day 1 of life for 5 days. Group 2 (n = 11): Hypoxia (HYP) Pups were subjected to hypoxia protocol (5% O_2_ and 95% N_2_ for 10 min) three times daily, beginning on day 1 of life for 5 days. Following each hypoxia episode, pups received IP injections of 100 µL NS. Group 3 (n = 21): Hypoxia-NAC (HYP-NAC) Pups were subject to hypoxia protocol and received NAC 300 mg/kg IP (SIGMA) reconstituted in water (total volume 100 µL) following each hypoxia episode. Group 4 (n = 23): NAC Hypoxia (NAC-HYP) included pups of dams treated with NAC during pregnancy and pups subject to hypoxia protocol and received IP injections of 100 µL NS following each hypoxia episode.

### 4.1. Experimental Procedures

#### 4.1.1. Neurological Function Evaluation

Righting reflex was assessed on the fifth day of life. Each pup was placed dorsal side down on a flat surface, then the latency to right onto all four paws was recorded. Latency to right was recorded three times for each group, and the average was used for statistical analysis [38].

#### 4.1.2. Sample Collection

On the fifth day of life pups were anesthetized with isoflurane, blood was collected from the heart and centrifuged at 4 °C to isolate serum for inflammation markers. Pups were decapitated and brains harvested and immediately frozen in liquid nitrogen or embedded in paraffin for further processing and analyses. Serum proinflammatory IL-6 protein levels were determined by ELISA and brain protein levels (NF-κB p65, neuronal nitric oxide synthase (nNOS), TNF-α, and IL-6) were determined by Western blot.

#### 4.1.3. ELISA Determinations

Commercial enzyme-linked immunosorbent assays (ELISA; R&D Systems, Minneapolis, MN, USA) kits were used to determine offspring serum protein levels of the IL-6 as previously described [20]. Fifty microliters of serum were needed for ELISA. For IL-6 assay, the minimum detectable concentration was 10 pg/mL; inter-assay and intra-assay variation was 10 percent. Maternal liver glutathione level was determined with a HT Glutathione Assay kit, catalog number: 7511-100-K (Bio-Techne Ltd., Minneapolis, MN, USA), according to the manufacturer’s instructions.

#### 4.1.4. Western Blot Analysis

Preparations of cell Lysates and Western blot cells or tissues were lysed in RIPA buffer (phosphate-buffered saline containing 1% NonidetP-40, 0.1% sodium dodecyl sulfate [SDS], 1 mM Na3VO1, 4 mM phenylmethylsulfonyl fluoride, and 0.05% [*w*/*v*] aprotinin). Insoluble proteins were discarded by high-speed centrifugation at 4 °C. A small volume of lysate was taken to perform protein estimation assay using absorbance at 280 nm in triplicate by Nanodrop. Protein sample concentrations were also compared to standards (BSA), ensuring that the standard was diluted into the same buffer as the samples, and then 50 μg of each sample were loaded in each well of a 12% SDS-PAGE acrylamide gel. After transferring and blocking to nitrocellulose membranes, the blots were incubated overnight at 4 °C, with the primary antibodies against the target protein. The antibodies were diluted in a blocking buffer according to the manufacturer’s recommended ratio. Following incubation with the primary antibodies and rinsing, the blots were incubated for 1 h at room temperature with HRP-conjugated secondary antibodies (according to the manufacturer’s recommended ratio-usually 1:10,000). Antibodies recognizing the nuclear factor kappa-light-chain-enhancer of activated B cells (NFkB) p65, active subunit (NB100-2176, Novus Biologicals, Centennial, CO, USA), neuronal nitric oxide synthase (nNOS) (NB120-3511, Novus Biologicals, Centennial, CO, USA), tumor necrosis factor-α (TNF-α) (NB600-587ss, Novus Biologicals, Centennial, CO, USA), and interleukin (IL)-6 (NB600-1131, Novus Biologicals, Centennial, CO, USA) were used with actin as the housekeeping gene. Densitometric analysis was used to determine differences in protein expression.

#### 4.1.5. Densitometric Analysis

We normalized the target protein expression levels to actin levels as our housekeeping protein. The quantification reflects the relative amounts, as a ratio of each protein band relative to the lanes of the control Actin. Films were subsequently imaged with ChemiDoc MP using the white light conversion screen and the silver stain (visible stain) application. The Band Analysis tools of ImageLab software version 4.1 (Bio-Rad, Haifa, Israel) were used to select and determine the background-subtracted density of the bands in all the gels and blots.

#### 4.1.6. Strip Blot Membranes

Blots were stripped and reprobed using Restore PLUS Western Blot Stripping Buffer (Catalog number: 46430) (Thermo Scientific, Cleveland, OH, USA).

#### 4.1.7. Terminal Deoxynucleotidyl Transferase dUTP Nick End Labeling (TUNEL)

Slides Preparation: Paraffin blocks were sectioned to approximately 3–5 microns thickness, put on a glass slide, stained with TUNEL Kit (ApopTag^®^ Peroxidase In Situ Apoptosis Detection Kit, Darmstadt, Germany) and covered by an automated machine. Embedding, sectioning, and stained slides preparation was performed by Patho-Logica, Ness Ziona, Israel.

Histology Evaluation: The histological analysis was outsourced by Dr. Emmanuel Loeb, the director of Pato-logical. The histology evaluation of all the slides was performed using an Olympus BX60 microscope, serial NO. 7D04032. All slides were examined by one pathologist (EL). Positive TUNEL reaction was presented as dark brown staining in the cell nuclei. Only neurons were scored for positive TUNEL reactions. All brain areas appearing on the slide were scanned. Areas of cerebral cortex, hippocampus, and white matter areas were evaluated. A scoring system (semi-quantitative) within a X40 magnification field was used as follows:

Grade 0: no cells with positive TUNEL stain.

Grade 1: 1–5 cells positive for TUNEL stain.

Grade 2: 6–20 cells positive for TUNEL stain.

Grade 3: >21 cells positive for TUNEL stain.

#### 4.1.8. Statistical Analysis

Offspring brain NFkB p65, nNOS, TNFα, IL-6, and serum IL-6 protein levels were compared among pups from the different groups using one-way analysis of variance followed by post hoc tests for pairwise comparisons (Holm-Sidak method). All results are expressed as means ± SD. Differences considered significant at *p <* 0.05.

## 5. Strengths and Limitations

The strength of our study is the established rat model that represents maternal treatment during pregnancy on controlled hypoxia insult on pups at day 1–5 of life. These controlled pre- and post-natal NAC effects during pregnancy and hypoxia mimic the effect on human brain development at the second/third trimester of pregnancy when the human brain is most vulnerable for long term sequelae [18]. We measured several pathways of injury and correlated markers with pathology and behavior. We recognize that apoptosis was evaluated on the fifth day of life, which may be too early to assess the final damage to neurons. Further studies and long-term studies are needed to define the protective effect of NAC. We have not determined the time course of increased brain markers, or how long before or after hypoxia can NAC be effective. NAC antioxidative and anti-inflammatory ability may cause fetal and maternal side effects although it is considered category B drug and considered safe to be used during pregnancy. Potential harmful effects have to be addressed in future studies.

We demonstrated for the first time that maternal NAC treatment can prevent hypoxia induced newborn brain injury. NAC administered to dams during pregnancy and to pups afterwards decreased both brain inflammatory cytokines and pathways associated with brain injury. Most interestingly, our novel findings that prophylactic NAC to dams during pregnancy confirms long-term protection to offspring hypoxia associated brain injury implies that we may consider prophylactic NAC treatment to patients at risk for hypoxia during labor. We prefer prophylaxis to dams rather than treatment to the offspring, since withholding NAC treatment after the hypoxia process has already started might be too late.

## Figures and Tables

**Table 1:** Pups weight and mortality in the different groups: Control, Hypoxia, NAC-Hypoxia and Hypoxia-NAC. * *p* value < 0.05 compered to Hypoxia.

	NAC-HYO (n = 23)	HYP-NAC (n = 21)	HYO (n = 11)	CON (n = 11)
	Birth Weight			
Average weight (gr)	6.26	5.94	6.07	6.16
SD	0.31	0.52	0.19	0.43
	Weight at day 5 of life			
Average (gr)	9.4 *	9.04	8.88	9.04
SD	0.74	0.66	0.21	0.32
	Mortality			
	2 (8.69%)	1 (4.76%)	2 (20%)	0 (0%)

## Data Availability

Derivative data supporting the findings of this study are available from the corresponding author upon request.

## Abbreviations

NAC	N-Acetyl Cysteine
nNOS	Neuronal nitric oxide synthase
IV	Intravenous
IP	Intraperitoneal
IL-6	Interleukin 6
NF-κB	Nuclear factor kappa-light-chain-enhancer of activated B cells
NS	Normal saline
TNFα	Tumor necrosis factor α.
